# Vonoprazan-based dual therapy with amoxicillin or doxycycline versus bismuth-containing quadruple therapy for *Helicobacter pylori* eradication: a prospective, multicenter, open-label, randomized controlled trial

**DOI:** 10.3389/fcimb.2026.1881347

**Published:** 2026-07-03

**Authors:** Haocheng Wang, Jun Wang, Lihong Shi, Guangxia Chen, Weixuan Yang, Chunming Fei, Xiaoli Huang, Zhimei Zhang, Xiujuan Wang, Jiahuan Gao, Zhenyu Zhang

**Affiliations:** 1 Department of Gastroenterology, Nanjing First Hospital, Nanjing Medical University, Nanjing, Jiangsu, China; 2 Department of Gastroenterology, Jinhu County People’s Hospital, Huaian, Jiangsu, China; 3 Department of Gastroenterology, The Second Affiliated Hospital, Xuzhou Medical University, Xuzhou, Jiangsu, China; 4 Department of Gastroenterology, Xuzhou First People’s Hospital, Xuzhou, Jiangsu, China; 5 Department of Gastroenterology, Huai’an Hospital Affiliated to Yangzhou University (The Fifth People’s Hospital of Huai’an), Huai’an, Jiangsu, China; 6 Department of Gastroenterology, The People’s Hospital of Luhe, Nanjing, Jiangsu, China; 7 Department of Gastroenterology, The Fourth Affiliated Hospital of Nanjing Medical University, Nanjing, Jiangsu, China; 8 Department of Gastroenterology, Lianyungang Clinical College of Nanjing Medical University, Lianyungang, Jiangsu, China

**Keywords:** amoxicillin, doxycycline, dual therapy, helicobacter pylori, vonoprazan

## Abstract

**Background:**

Bismuth-containing quadruple therapy, the standard *Helicobacter pylori* (*H. pylori*) eradication regimen, is limited by adverse events and rising resistance. Vonoprazan-amoxicillin dual therapy offers simplicity but has mostly been tested using high-dose amoxicillin. Data on doxycycline in this context are scarce, and vonoprazan-doxycycline dual therapy remains unexplored.

**Methods:**

A total of 579 *H. pylori*-positive patients were enrolled and randomly assigned in a 1:1:1 ratio to one of three 14-day treatment groups: the VBQ group (vonoprazan 20 mg, amoxicillin 1000 mg, doxycycline 100 mg, and colloidal bismuth pectin 300 mg, all twice daily); the VA group (vonoprazan 20 mg and amoxicillin 1000 mg, twice daily); and the VD group (vonoprazan 20 mg and doxycycline 100 mg, twice daily). A ^13^C-urea breath test (UBT) was performed at least four weeks after treatment completion. *H. pylori* eradication rates, the incidence of adverse events during treatment, and medication adherence were compared among the three groups.

**Results:**

In the intention-to-treat (ITT), modified intention-to-treat (mITT), and per-protocol (PP) analyses, the VA regimen demonstrated non-inferiority to the VBQ regimen. The VD regimen did not meet the non-inferiority criterion in the ITT analysis (79.3% vs. 83.4%, P = 0.070 for non-inferiority), but it demonstrated non-inferiority to the VBQ regimen in both the mITT (87.4% vs. 90.4%, P = 0.018) and PP (87.7% vs. 90.1%, P = 0.012) analyses. The overall incidence of adverse events was not significantly different between the VBQ and VA groups (P = 0.313), but it was significantly lower in the VD group compared to the VBQ group (P = 0.002). No statistically significant differences in medication adherence were observed between the VBQ group and either the VA (P = 0.381) or VD (P = 0.269) group.

**Conclusion:**

Vonoprazan-low-dose amoxicillin dual therapy is a safe and simple alternative to bismuth containing quadruple therapy. Vonoprazan-doxycycline dual therapy demonstrates acceptable efficacy and may be considered for patients allergic to amoxicillin. Both align with antimicrobial stewardship principles of regimen simplification.

## 1 Introduction

*Helicobacter pylori* (*H. pylori*) infection is a major cause of several diseases, including peptic ulcers, chronic gastritis, gastric mucosa-associated lymphoid tissue lymphoma, and gastric cancer (Malfertheiner et al., 2022), affecting approximately 44.2% of the population in mainland China (Ren et al., 2022). Although infection rates have declined in some developed countries, *H. pylori* remains a critical public health issue worldwide.

In China, the recommended first-line regimen for *H. pylori* eradication is bismuth-containing quadruple therapy. According to the *2022 Chinese National Clinical Practice Guideline on H. pylori Eradication Treatment* (Zhou et al., 2022), this regimen consists of a 14-day course including a proton pump inhibitor, bismuth, and two antibiotics selected from the following combinations: (1) amoxicillin 1.0 g plus clarithromycin 500 mg, twice daily; (2) amoxicillin 1.0 g twice daily plus levofloxacin 500 mg once daily or 200 mg twice daily; (3) tetracycline 500 mg (3–4 times daily) plus metronidazole 400 mg (3–4 times daily); (4) amoxicillin 1.0 g twice daily plus metronidazole 400 mg (3–4 times daily); (5) amoxicillin 1.0 g twice daily plus tetracycline 500 mg (3–4 times daily). However, the efficacy of this regimen has declined due to rising antibiotic resistance, particularly to clarithromycin, levofloxacin, and metronidazole (Savoldi et al., 2018). Additionally, quadruple therapy is associated with bismuth-related adverse effects and higher medication costs (Sun et al., 2025).

Dual therapy combining a single antibiotic with an acid suppressant has emerged as a promising alternative. Recent studies have shown that dual therapy can achieve eradication rates comparable to quadruple therapy with improved adherence and lower costs (Gao et al., 2020; Guan et al., 2022; Shen et al., 2022). Vonoprazan, a novel potassium-competitive acid blocker, offers potent and sustained acid suppression independent of CYP2C19 polymorphisms, enhancing antibiotic activity (Scarpignato and Hunt, 2019). Vonoprazan-amoxicillin dual therapy has shown high eradication rates and is a first-line regimen in Japan (Zuberi et al., 2022; Mori et al., 2019). Available data indicate that the per-protocol eradication rate for vonoprazan dual therapy exceeds 90%, offering further opportunities for regimen optimization (Hu et al., 2023). Although vonoprazan was approved for use in China in 2020, research on vonoprazan-based dual therapy in the Chinese population remains limited. Existing studies are mostly single-center and involve relatively homogeneous regimens, which may compromise the generalizability of their findings. Therefore, large-scale multicenter studies are needed to further validate the efficacy, reliability, and safety of vonoprazan-based dual therapy in the Chinese population.

Amoxicillin exhibits potent bactericidal activity and excellent efficacy in *H. pylori* eradication. Its extremely low resistance rate and favorable safety profile make it a preferred antibiotic for dual therapy (Duan et al., 2023). Vonoprazan-amoxicillin dual therapy is thus gaining increasing attention. The most commonly used regimen in clinical practice is a 14-day course combining a standard dose of vonoprazan (40 mg daily) with a high dose of amoxicillin (3000 mg daily). However, guided by antimicrobial stewardship principles, a key research focus has emerged on whether reducing the antibiotic dose can maintain eradication efficacy while further lowering costs, improving patient adherence, and reducing the incidence of adverse events (Hsu et al., 2025). A recent nationwide Chinese study demonstrated that low-dose amoxicillin (2 g/day) combined with vonoprazan was non-inferior to high-dose (3 g/day) therapy (Hu et al., 2025). Nevertheless, evidence is lacking on whether low-dose amoxicillin dual therapy is non-inferior to bismuth-containing quadruple therapy.

Amoxicillin is associated with adverse reactions, nearly half of which are allergic in nature, ranging from mild rash to anaphylactic shock (Liu and Nahata, 2023). For patients with amoxicillin allergy, alternative therapies are needed. Tetracycline has an antimicrobial spectrum similar to amoxicillin and an equally low resistance rate (Zhong et al., 2021). Doxycycline, a semisynthetic tetracycline derivative, offers greater antibacterial potency and a longer half-life than tetracycline, allowing for reduced dosing frequency and lower nephrotoxicity risk. A study has shown that a 14-day vonoprazan-tetracycline dual regimen is an effective and safe first-line treatment for penicillin-allergic patients with *H. pylor*i infection, achieving eradication rates exceeding 90% with a low incidence of adverse events (Gao et al., 2024). Another study reported that vonoprazan-tetracycline dual therapy demonstrated favorable efficacy in special populations, including penicillin-allergic patients or those with prior failure of amoxicillin-containing regimens, achieving an eradication rate of 93.5% (Gao et al., 2023). Therefore, a dual regimen combining doxycycline with vonoprazan may represent a reliable alternative for patients with amoxicillin allergy. Multicenter studies are warranted to further validate the efficacy, safety, and adherence of doxycycline-vonoprazan dual therapy. Compared to bismuth-containing quadruple therapy, dual regimens may offer a lower incidence of adverse events and reduced antibiotic exposure. Thus, a treatment strategy that demonstrates non-inferior efficacy while offering greater simplicity and an improved safety profile could serve as a valuable clinical alternative. Accordingly, this study aims to conduct a prospective, multicenter, non-inferiority randomized controlled trial comparing vonoprazan combined with low-dose amoxicillin (1.0 g twice daily) dual therapy, vonoprazan combined with doxycycline (1.0 g twice daily) dual therapy, and standard bismuth-containing quadruple therapy. By analyzing and comparing eradication rates, adverse events, and patient adherence across the three groups, this study seeks to provide safer, more effective, cost-efficient, and convenient eradication strategies for patients with *H. pylori* infection.

## 2 Materials and methods

### 2.1 Study overview

This study is a prospective, multicenter, open-label, non-inferiority randomized controlled trial. It was approved by the Medical Ethics Committee of Nanjing First Hospital Affiliated to Nanjing Medical University (Approval No. KY20240123-18), which served as the lead ethics committee. Additionally, the study protocol was approved by the institutional ethics committees of all participating centers. The study was registered with ClinicalTrials.gov (NCT06412588; registered on May 13, 2024). It was conducted in accordance with the Declaration of Helsinki, using commercially available and widely adopted medications. All participants were fully informed of potential risks and provided written informed consent.

### 2.2 Study participants

The study enrolled 579 patients diagnosed with *H. pylori* infection in the gastroenterology outpatient departments of Nanjing First Hospital, Jinhu County People’s Hospital, The Second Affiliated Hospital of Xuzhou Medical University, Xuzhou First People’s Hospital, The Fifth People’s Hospital of Huai’an, The People’s Hospital of Luhe, The Fourth Affiliated Hospital of Nanjing Medical University and Lianyungang Clinical College of Nanjing Medical University between June 15, 2024, and December 31, 2025.

#### 2.2.1 The inclusion criteria included

1. Age 18 to 70 years, inclusive;
2. Confirmed *H. pylori* infection by ^13^C- or ^14^C-urea breath test (UBT);
3. No prior history of *H. pylori* eradication treatment, or documented prior eradication failure with no eradication therapy within the preceding six months. Eradication failure was defined as a positive result for *H. pylori* on a UBT or other validated method at least four weeks after completion of a previous eradication regimen, with the medical records of prior failure verified;
4. Voluntary provision of written informed consent.

#### 2.2.2 The exclusion criteria were:

1. Allergy to any study medication (e.g., penicillin, amoxicillin, vonoprazan, doxycycline);
2. Confirmed active peptic ulcer disease;
3. Use of any *H. pylori* eradication therapy within the six months immediately preceding enrollment;
4. Use of antibiotics or bismuth agents within 4 weeks, or use of H_2_-receptor antagonists or PPIs within 2 weeks before treatment initiation;
5. Current use of corticosteroids, nonsteroidal anti-inflammatory drugs, anticoagulants, barbiturates, phenytoin, or carbamazepine;
6. History of esophageal or gastric surgery;
7. Pregnancy or lactation;
8. Alcohol abuse;
9. Severe comorbidities, such as hepatic, cardiovascular, pulmonary, or renal disease;
10. Hepatic insufficiency due to hepatitis, fatty liver, or other causes;
11. Gastric mucosa-associated lymphoid tissue lymphoma or other malignancies.

### 2.3 Sample size calculation

Based on previously published efficacy data for bismuth-containing quadruple therapy, vonoprazan-amoxicillin dual therapy, and vonoprazan-tetracycline dual therapy, as well as our preliminary results, we assumed an eradication rate of 92% for the bismuth-containing quadruple (VBQ) group, 91% for the vonoprazan-amoxicillin (VA) group, and 91% for the vonoprazan-doxycycline (VD) group. To establish non-inferiority of the VA or VD regimen compared to the VBQ regimen, a non-inferiority margin of -10% was set, with a one-sided significance level of 0.025. To account for two non-inferiority comparisons, the significance level was adjusted to 0.0125 one-sided. To achieve 80% power, a sample size calculation performed using R software yielded a requirement of 183 participants per group. Anticipating a 5% loss to follow-up or refusal rate, a minimum of 193 participants per group was required. Based on this calculation and considering recruitment feasibility across clinical centers, a total of 579 participants (193 per group) were enrolled in this study.

The choice of a -10% non-inferiority margin was based on both clinical and statistical considerations. From a clinical perspective, a -10% margin was considered acceptable. Previous high-quality randomized controlled trials had demonstrated that when the loss of efficacy was limited to within 10%, the advantages of dual therapy including fewer medications, reduced antibiotic exposure, and a lower expected risk of adverse events were sufficient to offset this small difference (Qian et al., 2023). Although a 10% margin may seem relatively wide when the expected eradication rate in the control group is as high as 92%, a reduction from 92% to 82% in actual practice still exceeds the generally accepted 80% threshold for clinical practice guidelines. This is especially true when the latter is achieved with fewer medications, lower costs, and improved safety. From a statistical perspective, the -10% margin was selected to ensure that the lower limit of the 95% confidence interval for the eradication rate of dual therapy remains above 80%. The 80% threshold represents a clinically meaningful benchmark, as eradication rates below 80% are typically considered unsatisfactory in *H. pylori* treatment guidelines. By setting the margin at -10%, we ensured that even in the worst-case scenario, the dual therapy regimen would achieve an eradication rate of no less than 80%, which is clinically acceptable.

### 2.4 Randomization procedure

Randomization was performed using a block design with a block size of 6. A professional biostatistician generated the randomization sequence using SAS software (version 9.3). Eligible participants were assigned with equal probability in a 1:1:1 ratio to the VBQ, VA, or VD group. After providing informed consent, eligible patients with *H. pylori* infection completed a baseline information questionnaire on the Wenjuanxing platform. The platform backend matched participants to the pre-generated randomization sequence based on the order of questionnaire submission. Upon submission of the baseline questionnaire, the assigned group was displayed directly on the participant’s mobile device. Investigators then administered the corresponding treatment regimen according to this group assignment.

To ensure rigorous randomization and effective allocation concealment, the randomization sequence generated by a professional biostatistician was uploaded to the backend of the Wenjuanxing platform and concealed from all investigators and site staff, who had no access to the sequence. The platform’s backend system assigned participants strictly in accordance with the sequence order; allocations could not be skipped, rearranged, or modified, and each allocation was timestamped. Although the assigned group was displayed on the participant’s device, this information was generated only after the participant submitted the questionnaire; investigators had no prior knowledge of or ability to influence the allocation before assignment. In addition, randomization was stratified by study center to ensure a balanced distribution of the three treatment groups within each center.

### 2.5 Study procedures

#### 2.5.1 Intervention and grouping

Eligible participants were randomized 1:1:1 into three treatment groups using the method described above: the bismuth-containing quadruple therapy (VBQ) group, the vonoprazan-amoxicillin dual therapy (VA) group, and the vonoprazan-doxycycline dual therapy (VD) group. The VBQ group received a 14-day regimen consisting of vonoprazan fumarate tablets (Vocinti^®^, 20 mg/tablet, manufactured by Takeda Pharmaceutical Company Limited) 20 mg twice daily, amoxicillin capsules (Amoxil^®^, 250 mg/capsule, manufactured by Zhuhai United Pharmaceutical Co., Ltd., China) 1.0 g twice daily, doxycycline tablets (Xiyan^®^, 0.1 g/tablet, manufactured by Jiangsu Lianhuan Pharmaceutical Co.,Ltd., China) 0.1 g twice daily, and colloidal bismuth pectin for suspension (Huanafor^®^, 150 mg/sachet, manufactured by Hunan Warrant Pharmaceutical Co., Ltd., China) 0.3 g twice daily. The VA group received a 14-day regimen of vonoprazan fumarate tablets (20 mg twice daily) and amoxicillin (1.0 g twice daily). The VD group received a 14-day regimen of vonoprazan fumarate tablets (20 mg twice daily) and doxycycline (0.1 g twice daily). Vonoprazan and colloidal bismuth pectin were taken 30 minutes before breakfast and dinner, while amoxicillin and doxycycline were taken 30 minutes after breakfast and dinner. Participants were instructed to take all medications with plain water and to avoid carbonated beverages, strong tea, coffee, acidic juices, and dairy products around the time of drug intake. Alcohol consumption and smoking were restricted from one day before treatment until three days after treatment completion. Additionally, the use of acid suppressants, antibiotics, bismuth preparations, herbal products with antibacterial properties, and other probiotics was prohibited from enrollment until the follow-up assessment.

#### 2.5.2 Data collection and follow-up

Baseline data were collected from each participant prior to treatment initiation using an electronic questionnaire, which covered demographic and clinical characteristics. To minimize recall bias, we collected data on participants’ antibiotic use over the previous two years using a visual-assisted recall method. A color-illustrated booklet was developed, containing the names and images of commonly available antibiotics on the market (including amoxicillin/penicillin, clarithromycin, metronidazole/tinidazole, levofloxacin, furazolidone, tetracycline, cephalosporins, etc.). At enrollment, participants were systematically shown the images and names of each antibiotic class and asked to identify those they had used in the preceding two years.

During the 14-day eradication therapy, participants were provided with a pre-designed paper record form and instructed to document daily on this form any adverse events experienced and the number of doses of each medication taken each day. Participants were asked to complete the record form in real time on a daily basis throughout the treatment period. Follow-up assessments were scheduled at two time points: within 3 days after treatment completion and at least 4 weeks post-treatment. Within 3 days after completing the regimen, study staff contacted participants via online platforms or telephone to collect the completed record forms. During this follow-up, participants reported the information recorded in the forms, including daily medication adherence and any adverse events that had occurred during the treatment period. Medication adherence was calculated as the proportion of prescribed doses actually taken based on the information recorded in the forms. Adherence of at least 80% was considered good, while adherence below 80% was defined as poor. At the follow-up visit at least 4 weeks after treatment completion, participants returned to the clinic for a ^13^C-UBT to determine *H. pylori* eradication status, with a DOB value <4 defined as successful eradication.

#### 2.5.3 Outcome measures

(1)Primary outcome: *H. pylori* eradication rate.

Eradication success was defined as a negative ^13^C-UBT result conducted at least four weeks after treatment completion.

(2)Secondary outcomes: Medication adherence and incidence of adverse events.

Medication adherence was measured as the proportion of prescribed doses actually taken during the 14-day treatment period, calculated based on the information recorded by participants in the pre-designed record forms. Adherence of at least 80% was considered good, while adherence below 80% was defined as poor. The incidence of adverse events was also documented based on the information recorded in the same forms.

### 2.6 Statistical analysis

The primary outcome was evaluated using three analysis sets: intention-to-treat (ITT), modified intention-to-treat (mITT), and per-protocol (PP). The ITT set included all randomized participants, regardless of whether they took the study medication or completed follow-up; participants with missing UBT outcomes were considered treatment failures. The mITT set comprised all randomized participants who took the assigned medication and completed a valid urea breath test follow-up, regardless of adherence level. The PP set included participants who completed a valid urea breath test and demonstrated good medication adherence (≥80% of prescribed doses).

For continuous variables, comparisons were performed using the independent samples t-test, and data were presented as mean ± standard deviation. For categorical variables, comparisons were performed using the chi-square test or Fisher’s exact test, and data were presented as frequencies and proportions.

Non-inferiority of the VA or VD regimen compared to the VBQ regimen was assessed using a non-inferiority Z-test based on normal approximation. The non-inferiority P-value was calculated as a one-sided P-value. The null hypothesis was that the eradication rate of the experimental regimen (VA or VD) was at least 10% lower than that of the reference regimen (VBQ), and the alternative hypothesis was that the difference was less than 10%. The 95% confidence intervals for the risk difference (experimental group minus control group) were calculated using the Newcombe-Wilson method. Non-inferiority was concluded if both of the following criteria were met: (1) the one-sided non-inferiority P-value was less than the pre-specified significance level (0.025 for a single comparison); (2) the lower bound of the two-sided 95% confidence interval for the risk difference was greater than the pre-specified non-inferiority margin of -10%.

Non-inferiority testing in this study was performed using SAS software (version 9.4, SAS Institute Inc.). All other statistical analyses were conducted using SPSS software (version 27.0). A P-value <0.05 was considered statistically significant.

## 3 Results

### 3.1 Participant enrollment

**Figure 1:**
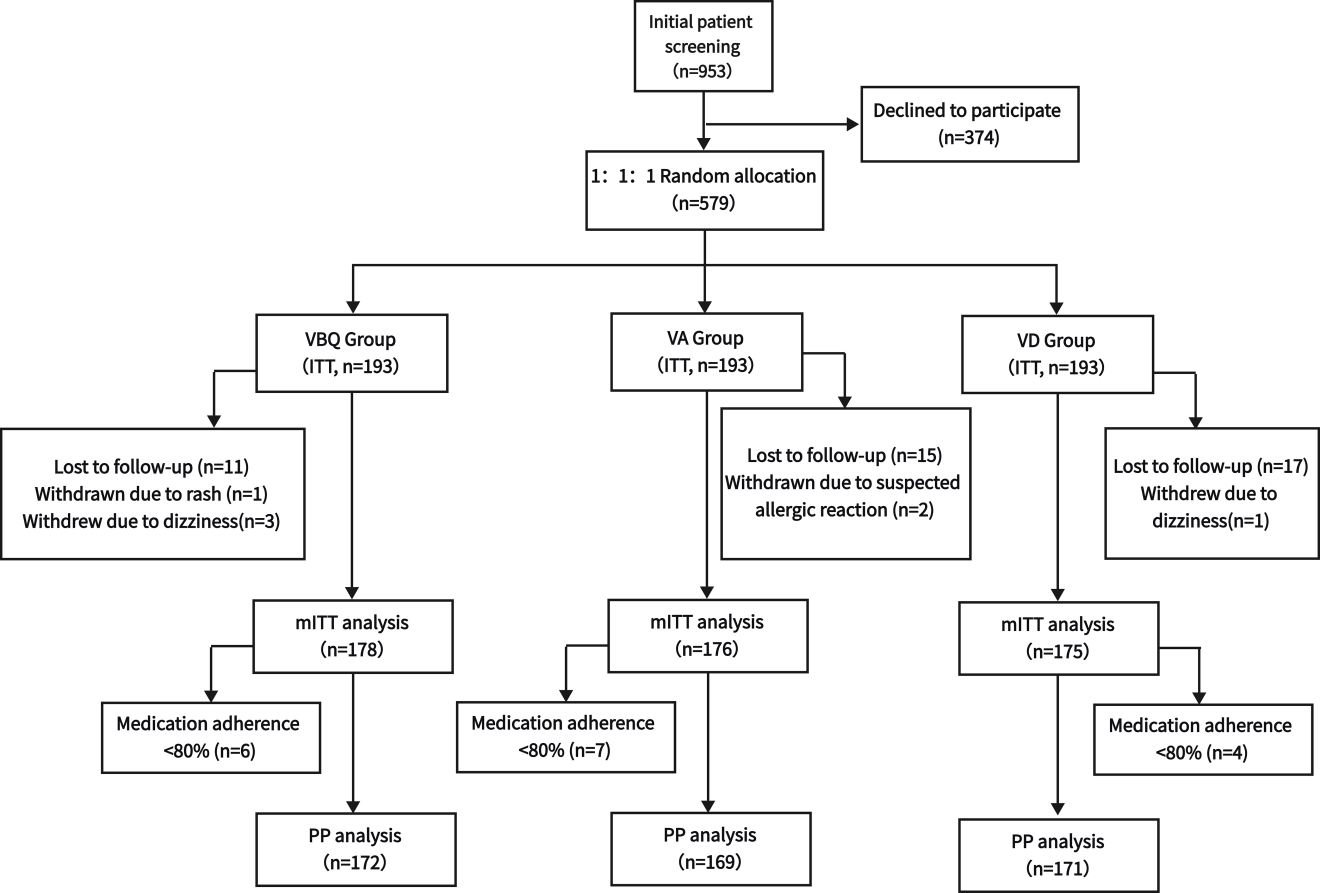
Study flow diagram.

Between June 15, 2024, and December 31, 2025, a total of 953 patients were screened, of whom 579 eligible patients were enrolled and randomly assigned equally to the VBQ, VA, and VD groups (193 patients each). The ITT analysis included all 579 randomized patients. After excluding 50 patients who were lost to follow-up or withdrew, 529 patients were included in the mITT analysis (178 in VBQ, 176 in VA, 175 in VD). After further excluding 17 patients with poor adherence (<80%), 512 patients were included in the PP analysis (172 in VBQ, 169 in VA, 171 in VD). The study flow is detailed in **Figure 1**.

### 3.2 Baseline characteristics

The baseline and clinical characteristics of the 579 included participants are presented in **Table 1**. In the VBQ group, there were 93 men (48.2%) and 100 women (51.8%), with a mean age of 49.55 ± 11.64 years and a mean body mass index (BMI) of 24.34 ± 3.37 kg/m². The VA group comprised 84 men (43.5%) and 109 women (56.5%), with a mean age of 46.76 ± 12.95 years and a mean BMI of 23.24 ± 3.04 kg/m². The VD group consisted of 102 men (52.8%) and 91 women (47.2%), with a mean age of 48.25 ± 12.67 years and a mean BMI of 23.31 ± 3.33 kg/m². No statistically significant differences were observed among the three groups in terms of sex, age, history of comorbidities, alcohol use, prior eradication therapy, or history of antibiotic use within the past two years (all P>0.05). However, significant differences were found in BMI (P = 0.002), smoking history (P = 0.012), and the presence of gastrointestinal symptoms within the past three months (P = 0.001).

**Table 1:** Baseline demographic and clinical characteristics of the enrolled participants.

Variables	VBQ group (n=193)	VA group (n=193)	VD group (n=193)	P-value
Sex (Male/Female)	93/100	84/109	102/91	0.186
Age (years)	49.55 ± 11.64	46.76 ± 12.95	48.25 ± 12.67	0.095
BMI (kg/m^2^)	24.34 ± 3.37	23.24 ± 3.04	23.31 ± 3.33	0.002
Medical history				
Hypertension	35 (18.1%)	24 (12.4%)	39 (20.2%)	0.108
Diabetes	13 (6.7%)	10 (5.2%)	16 (8.3%)	0.476
Hyperlipidemia	17 (8.8%)	10 (5.2%)	15 (7.8%)	0.368
Coronary Heart Disease	1 (0.5%)	2 (1.0%)	6 (3.1%)	0.093
Other	15 (7.8%)	24 (12.4%)	26 (13.5%)	0.168
Smoking history	59 (30.6%)	37 (19.2%)	60 (31.1%)	0.012
Alcohol consumption history	85 (44.0%)	64 (33.2%)	81 (42.0%)	0.068
Previous eradication therapy	16 (8.3%)	8 (4.1%)	9 (4.7%)	0.160
Recent Gastrointestinal Symptoms	63 (32.6%)	78 (40.4%)	100 (51.8%)	0.001
Antibiotic use in past 2 years				
Amoxicillin/Penicillin	57 (29.5%)	52 (26.9%)	59 (30.6%)	0.721
Clarithromycin	10 (5.2%)	9 (4.7%)	11 (5.7%)	0.900
Metronidazole/Tinidazole	9 (4.7%)	7 (3.6%)	14 (7.3%)	0.254
Levofloxacin	29 (15.0%)	17 (8.8%)	27 (14.0%)	0.143
Furazolidone	2 (1.0%)	0	1 (0.5%)	0.366
Tetracycline	1 (0.5%)	0	1 (0.5%)	0.605
Cephalosporin	67 (34.7%)	55 (28.5%)	53 (27.5%)	0.244
Other/Unknown	16 (8.3%)	20 (10.4%)	24 (12.4%)	0.410

VBQ, vonoprazan-bismuth-amoxicillin-doxycycline quadruple therapy; VA, vonoprazan-amoxicillin dual therapy; VD, vonoprazan-doxycycline dual therapy; BMI, body mass index; *H. pylori*, *Helicobacter pylori*.

### 3.3 Comparison of *H. pylori* eradication rates among groups

#### 3.3.1 VBQ group vs. VA group

The eradication rates for the VBQ and VA groups are presented in **Table 2**.

**Table 2:** Non-inferiority comparison of *H. pylori* eradication rates between the VA and VBQ groups.

Analysis set	VBQ	VA	P-value for superiority	P-value for non-inferiority	Risk difference*
ITT	83.4% (161/193) (95% CI: 78.2%, 88.7%)	82.4% (159/193) (95% CI: 77.0%, 87.8%)	0.787	0.010	-1.04% (95% CI: -8.55%, 6.47%)
mITT	90.4% (161/178) (95% CI: 86.1%, 94.8%)	90.3% (159/176) (95% CI: 86.0%, 94.7%)	0.972	<0.001	-0.11% (95% CI: -6.25%, 6.03%)
PP	90.1% (155/172) (95% CI: 85.7%, 94.6%)	90.5% (153/169) (95% CI: 86.1%, 94.9%)	0.897	<0.001	0.42% (95% CI: -5.86%, 6.69%)

*Risk difference calculated as VA group minus VBQ group. The prespecified non-inferiority margin was -10%. Non-inferiority is concluded if the lower limit of the 95% confidence interval for the risk difference is above -10%.

*H. pylori*, *Helicobacter pylori*; VBQ, vonoprazan-bismuth-amoxicillin-doxycycline quadruple therapy; VA, vonoprazan-amoxicillin dual therapy; ITT, intention-to-treat; mITT, modified intention-to-treat; PP, per-protocol.

In the primary mITT analysis, the VA regimen achieved an eradication rate of 90.3%, which was non-inferior to the VBQ regimen (90.4%), with a risk difference of -0.11% (95% CI: -6.25% to 6.03%) and a non-inferiority P-value <0.001. No statistically significant difference was observed between the two regimens (P = 0.972). Consistent results were observed in the ITT and PP analyses. In the ITT analysis, the eradication rate was 82.4% for VA and 83.4% for VBQ, with non-inferiority established (P = 0.010). In the PP analysis, the eradication rate was 90.5% for VA and 90.1% for VBQ, with non-inferiority also established (P<0.001).

In summary, the eradication rate in the VA group was non-inferior to that in the VBQ group.

#### 3.3.2 VBQ group vs. VD group

The eradication rates for the VBQ and VD groups are presented in **Table 3**.

**Table 3:** Non-inferiority comparison of *H. pylori* eradication rates between the VD and VBQ groups.

Analysis set	VBQ	VD	P-value for superiority	P-value for non-inferiority	Risk difference*
ITT	83.4% (161/193) (95% CI: 78.2%, 88.7%)	79.3% (153/193) (95% CI: 73.6%, 85.0%)	0.296	0.070	-4.15% (95% CI: -11.91%, 3.61%)
mITT	90.4% (161/178) (95% CI: 86.1%, 94.8%)	87.4% (153/175) (95% CI: 82.5%, 92.3%)	0.365	0.018	-3.02% (95% CI: -9.56%, 3.52%)
PP	90.1% (155/172) (95% CI: 85.7%, 94.6%)	87.7% (150/171) (95% CI: 82.8%, 92.6%)	0.479	0.012	-2.40% (95% CI: -9.04%, 4.24%)

*Risk difference calculated as VD group minus VBQ group. The prespecified non-inferiority margin was -10%. Non-inferiority is concluded if the lower limit of the 95% confidence interval for the risk difference is above -10%.

*H. pylori*, *Helicobacter pylori*; VBQ, vonoprazan-bismuth-amoxicillin-doxycycline quadruple therapy; VD, vonoprazan-doxycycline dual therapy; ITT, intention-to-treat; mITT, modified intention-to-treat; PP, per-protocol.

In the primary mITT analysis, the VD regimen achieved an eradication rate of 87.4%, which was non-inferior to the VBQ regimen (90.4%), with a risk difference of -3.02% (95% CI: -9.56% to 3.52%) and a non-inferiority P-value of 0.018. No statistically significant difference was observed between the two regimens (P = 0.365). In the PP analysis, the VD regimen also demonstrated non-inferiority (87.7% vs. 90.1%; risk difference -2.40%, 95% CI: -9.04% to 4.24%; non-inferiority P = 0.012). However, in the ITT analysis, the non-inferiority criterion was not met (P = 0.070).

In summary, the eradication rate in the VD group was non-inferior to that in the VBQ group in both the mITT and PP analyses.

#### 3.3.3 Subgroup analysis of eradication rates based on prior treatment history

Subgroup analyses were performed according to patients’ prior treatment history. Subgroup analyses comparing the VBQ and VA groups are summarized in **Table 4**. In the treatment-naïve subgroup, the primary mITT analysis showed a risk difference of 2.16% (95% CI: -4.14% to 8.46%) between VA and VBQ. In the rescue therapy subgroup, the mITT analysis showed a risk difference of -37.50% (95% CI: -71.05% to -3.95%). Consistent results were observed in the ITT and PP analyses.

**Table 4:** Subgroup analysis of eradication rates between the VA and VBQ groups in treatment-naïve and rescue therapy patients.

Analysis set	Subgroup	VBQ	VA	P-value for superiority	P-value for non-inferiority	Risk difference*
ITT	Treatment-naïve	81.9% (145/177) (95% CI: 76.3%, 87.6%)	83.2% (154/185) (95% CI: 77.9%, 88.6%)	0.740	0.002	1.32% (95% CI: -6.49%, 9.14%)
	Rescue therapy	100.0% (16/16) (95% CI: 79.4%, 100.0%)	62.5% (5/8) (95% CI: 29.0%, 96.0%)	0.009	0.054	-37.50% (95% CI: -71.05%, -3.95%)
mITT	Treatment-naïve	89.5% (145/162) (95% CI: 84.8%, 94.2%)	91.7% (154/168) (95% CI: 87.5%, 95.8%)	0.501	<0.001	2.16% (95% CI: -4.14%, 8.46%)
	Rescue therapy	100.0% (16/16) (95% CI: 79.4%, 100.0%)	62.5% (5/8) (95% CI: 29.0%, 96.0%)	0.009	0.054	-37.50% (95% CI: -71.05%, -3.95%)
PP	Treatment-naïve	89.2% (140/157) (95% CI: 84.3%, 94.0%)	91.9% (149/162) (95% CI: 87.8%, 96.2%)	0.391	<0.001	2.80% (95% CI: -3.61%, 9.22%)
	Rescue therapy	100.0% (15/15) (95% CI: 78.2%, 100.0%)	57.1% (4/7) (95% CI: 20.5%, 93.8%)	0.006	0.040	-42.86% (95% CI: -79.52%, -6.20%)

*Risk difference calculated as VA group minus VBQ group. The prespecified non-inferiority margin was -10%. Non-inferiority is concluded if the lower limit of the 95% confidence interval for the risk difference is above -10%.

*H. pylori*, *Helicobacter pylori*; VBQ, vonoprazan-bismuth-amoxicillin-doxycycline quadruple therapy; VA, vonoprazan-amoxicillin dual therapy; ITT, intention-to-treat; mITT, modified intention-to-treat; PP, per-protocol.

In addition, subgroup analyses comparing the VBQ and VD groups are summarized in **Table 5**. In the treatment-naïve subgroup, the primary mITT analysis showed a risk difference of -2.16% (95% CI: -9.07% to 4.76%) between VD and VBQ. In the rescue therapy subgroup, the mITT analysis showed a risk difference of -11.11% (95% CI: -31.64% to 9.42%). Consistent results were observed in the ITT and PP analyses.

**Table 5:** Subgroup analysis of eradication rates between the VD and VBQ groups in treatment-naïve and rescue therapy patients.

Analysis set	Subgroup	VBQ	VD	P-value for superiority	P-value for non-inferiority	Risk difference*
ITT	Treatment-naïve	81.9% (145/177) (95% CI: 76.3%, 87.6%)	78.8% (145/184) (95% CI: 72.9%, 84.7%)	0.456	0.050	-3.12% (95% CI: -11.30%, 5.07%)
mITT	Rescue therapy	100.0% (16/16) (95% CI: 79.4%, 100.0%)	88.9% (8/9) (95% CI: 68.4%, 100.0%)	0.174	0.458	-11.11% (95% CI: -31.64%, 9.42%)
	Treatment-naïve	89.5% (145/162) (95% CI: 84.8%, 94.2%)	87.3% (145/166) (95% CI: 82.3%, 92.4%)	0.542	0.013	-2.16% (95% CI: -9.07%, 4.76%)
	Rescue therapy	100.0% (16/16) (95% CI: 79.4%, 100.0%)	88.9% (8/9 (95% CI: 68.4%, 100.0%)	0.174	0.458	-11.11% (95% CI: -31.64%, 9.42%)
PP	Treatment-naïve	89.2% (140/157) (95% CI: 84.3%, 94.0%)	87.7% (142/162) (95% CI: 82.6%, 92.7%)	0.672	0.009	-1.52% (95% CI: -8.54%, 5.50%)
	Rescue therapy	100.0% (15/15) (95% CI: 78.2%, 100.0%)	88.9% (8/9) (95% CI: 68.4%, 100.0%)	0.188	0.458	-11.11% (95% CI: -31.64%, 9.42%)

*Risk difference calculated as VD group minus VBQ group. The prespecified non-inferiority margin was -10%. Non-inferiority is concluded if the lower limit of the 95% confidence interval for the risk difference is above -10%.

*H. pylori*, *Helicobacter pylori*; VBQ, vonoprazan-bismuth-amoxicillin-doxycycline quadruple therapy; VD, vonoprazan-doxycycline dual therapy; ITT, intention-to-treat; mITT, modified intention-to-treat; PP, per-protocol.

### 3.4 Analysis of adverse effects and adherence

Adverse events and medication adherence are summarized in **Table 6**. The overall incidence of adverse events was 20.2% (36/178) in the VBQ group, 18.2% (32/176) in the VA group, and 9.1% (16/175) in the VD group. There was no significant difference between the VBQ and VA groups (P = 0.626), while the VD group had a significantly lower incidence than the VBQ group (9.1% vs. 20.2%, P = 0.003).

**Table 6:** Analysis of adverse effects and medication adherence.

Adverse events	VBQ (n=178)	VA (n=176)	VD (n=175)	P-value^a^	P-value^b^
Adverse effects					
Overall incidence	36 (20.2%)	32 (18.2%)	16 (9.1%)	0.626	0.003
Diarrhea	4 (2.2%)	3 (1.7%)	2 (1.1%)	0.714	0.422
Dizziness	2 (1.1%)	3 (1.7%)	3 (1.7%)	0.643	0.639
Acid reflux/heartburn	10 (5.6%)	8 (4.5%)	2 (1.1%)	0.646	0.020
Nausea/vomiting	7 (3.9%)	6 (3.4%)	6 (3.4%)	0.794	0.802
Abdominal bloating	11 (6.2%)	10 (5.7%)	3 (1.7%)	0.843	0.032
Abdominal pain	7 (3.9%)	2 (1.1%)	1 (0.6%)	0.095	0.034
Rash	3 (1.7%)	1 (0.6%)	0 (0.0%)	0.320	0.085
Bad breath	2 (1.1%)	2 (1.1%)	1 (0.6%)	1.000	0.572
Other	2 (1.1%)	4 (2.3%)	0 (0.0%)	0.402	0.160
Good medication adherence	172 (96.6%)	169 (96.0%)	171 (97.7%)	0.762	0.539

^a^ VA group vs. VBQ group; ^b^VD group vs. VBQ group.

VBQ, vonoprazan-bismuth-amoxicillin-doxycycline quadruple therapy; VA, vonoprazan-amoxicillin dual therapy; VD, vonoprazan-doxycycline dual therapy.

Compared to the VBQ group, the VD group showed significantly lower rates of acid reflux/heartburn (1.1% vs. 5.6%, P = 0.020), abdominal bloating (1.7% vs. 6.2%, P = 0.032), and abdominal pain (0.6% vs. 3.9%, P = 0.034). No other significant differences in specific adverse events were observed between groups.

Medication adherence exceeded 96% in all three groups (VBQ: 96.6%, VA: 96.0%, VD: 97.7%), with no significant differences between the VBQ group and either the VA group (P = 0.762) or the VD group (P = 0.539).

### 3.5 Regression analysis of factors influencing *H. pylori* eradication rates

Univariate logistic regression analysis was performed to assess the influence of baseline characteristics, clinical features, and adverse events on *H. pylori* eradication rates. Using a negative retest result as the dependent variable in the PP population, factors including sex, age, BMI, history of comorbidities, smoking and alcohol use, prior eradication therapy, recent gastrointestinal symptoms, history of antibiotic use, and occurrence of adverse events were analyzed. As shown in **Table 7**, no significant associations with eradication outcome were found for sex, age, BMI, history of comorbidities, smoking and alcohol use, prior eradication therapy, recent gastrointestinal symptoms, or occurrence of adverse events. Among the antibiotic use history variables, only a history of cephalosporin use was identified as a protective factor against *H. pylori* eradication failure (OR = 0.467, 95% CI: 0.228-0.953, P = 0.036). No other antibiotic use history showed a significant association with eradication rate.

**Table 7:** Regression analysis of factors influencing *H. pylori* eradication rates.

Variables	Eradication success (n=458)	Eradication failure (n=54)	P-value
Sex (Male/Female)	221/237	22/32	0.297
Age (years)	48.57 ± 12.62	49.43 ± 11.44	0.633
BMI (kg/m^2^)	23.65 ± 3.34	23.91 ± 3.34	0.589
Medical history			
Hypertension	84 (18.3%)	6 (11.1%)	0.193
Diabetes	31 (6.8%)	6 (11.1%)	0.248
Hyperlipidemia	34 (7.4%)	3 (5.6%)	0.617
Coronary Heart Disease	8 (1.7%)	0	0.622
Other	53 (11.6%)	7 (13.0%)	0.764
Smoking history	120 (26.2%)	12 (22.2%)	0.528
Alcohol consumption	173 (37.8%)	20 (37.0%)	0.914
Previous eradication therapy	27 (5.9%)	4 (7.4%)	0.660
Recent Gastrointestinal Symptoms	197 (43.0%)	19 (35.2%)	0.273
Antibiotic use in past 2 years			
Amoxicillin/Penicillin	129 (28.2%)	17 (31.5%)	0.609
Clarithromycin	23 (5.0%)	2 (3.7%)	0.672
Metronidazole/Tinidazole	24 (5.2%)	1 (1.9%)	0.297
Levofloxacin	60 (13.1%)	7 (13.0%)	0.978
Furazolidone	2 (0.4%)	0	0.740
Tetracycline	2 (0.4%)	0	0.740
Cephalosporin	150 (32.8%)	10 (18.5%)	0.036
Other/Unknown	50 (10.9%)	7 (13.0%)	0.651
Adverse Effects	65 (14.2%)	10 (18.5%)	0.397

H. pylori, Helicobacter pylori; BMI, body mass index.

Multivariable logistic regression analysis was performed using a negative retest result in the per-protocol population as the dependent variable, with all variables from the univariate analysis entered into the model. The results showed that none of the variables reached statistical significance, with all P-values exceeding 0.05.

## 4 Discussion

Currently, bismuth-containing quadruple therapy is the first-line regimen for *H. pylori* eradication in China. However, concerns regarding its associated adverse events, suboptimal patient adherence, and rising antibiotic resistance rates are becoming increasingly prominent (Hu et al., 2020; Zhou et al., 2022). In recent years, vonoprazan-amoxicillin dual therapy has garnered widespread attention due to its simplicity and favorable eradication rates. Nevertheless, previous studies have predominantly employed amoxicillin regimens administered three or four times daily, involving high dosing frequencies and often a total daily dose of 3 g. Such regimens may compromise patient adherence, potentially increase the risk of adverse events, and contribute to the development of resistance (Liu et al., 2024). Furthermore, research on the use of doxycycline, a tetracycline antibiotic, in *H. pylori* eradication therapy remains limited. Doxycycline is currently used primarily as a component of quadruple regimens, and its efficacy and safety when combined with vonoprazan as a dual therapy have not been reported. Therefore, systematically comparing and evaluating the performance of low-dose amoxicillin and doxycycline in *H. pylori* dual therapy holds significant practical importance for optimizing clinical treatment choices.

This study is the first to evaluate the efficacy and safety of vonoprazan-based dual therapy with low-dose amoxicillin and with doxycycline for *H. pylori* eradication, and to compare these regimens non-inferiorly with traditional bismuth-containing quadruple therapy. The results demonstrated that a 14-day regimen of vonoprazan combined with low-dose amoxicillin achieved eradication rates non-inferior to those of bismuth quadruple therapy, consistent with findings by Hu et al (Hu et al., 2025). Amoxicillin, a semisynthetic penicillin, is characterized by an extremely low resistance rate, a favorable safety profile, and time-dependent bactericidal activity, theoretically requiring multiple daily doses to maintain effective plasma concentrations (de Velde et al., 2016). However, vonoprazan, through its potent and sustained acid suppression, significantly elevates intragastric pH, thereby enhancing the stability and prolonging the contact time of amoxicillin in the stomach. This may reduce the need for high-frequency dosing. Accordingly, the findings of this study confirm that, compared to previously reported high-frequency regimens, this simplified strategy of vonoprazan combined with low-dose amoxicillin dual therapy achieves satisfactory clinical efficacy while potentially improving patient adherence and reducing the risk of resistance.

Although the 2024 American College of Gastroenterology clinical guideline does not recommend substituting doxycycline for tetracycline in bismuth-containing quadruple therapy, a recommendation primarily based on the lack of clinical evidence and limited data suggesting suboptimal eradication efficacy (Chey et al., 2024), the clinical use of tetracycline itself is facing increasing challenges. As an antibiotic with a low resistance rate, tetracycline should theoretically be an important option for *H. pylori* eradication. However, its clinical application has progressively declined. Many healthcare facilities, particularly primary care hospitals, encounter difficulties in procuring tetracycline, creating practical barriers for patients in accessing the medication (Jiang et al., 2021). Furthermore, adverse effects associated with tetracycline, such as photosensitivity, vestibular toxicity, and renal effects, have also limited its widespread use (Montgomery and Worswick, 2022; Hamilton and Guarascio, 2019). Doxycycline, a tetracycline derivative, shares the same antibacterial mechanism as tetracycline but offers distinct pharmacological advantages. Studies have shown that doxycycline has greater antibacterial activity than tetracycline and a longer half-life, allowing for once- or twice-daily dosing, which can improve patient adherence. Additionally, doxycycline carries a lower risk of nephrotoxicity compared to tetracycline, making it a safer option for patients with renal impairment (Singh et al., 2021). Research from other countries has demonstrated extremely low resistance rates of *H. pylori* to doxycycline, suggesting its potential value in *H. pylori* eradication (Kouitcheu Mabeku et al., 2019).

Our study results showed that the doxycycline-containing bismuth quadruple regimen achieved eradication rates exceeding 90% in both the mITT and PP analyses, indicating satisfactory efficacy. This may be attributable to the use of vonoprazan for acid suppression in this study. As a novel potassium-competitive acid blocker, vonoprazan provides more potent and sustained gastric acid inhibition, creating a favorable environment for the antibacterial activity of both doxycycline and amoxicillin. More importantly, this study is the first to demonstrate that the eradication rate of vonoprazan-doxycycline dual therapy was non-inferior to that of bismuth-containing quadruple therapy in the mITT and PP analyses. However, it should be noted that the VD regimen achieved an eradication rate of 87.4% in the mITT analysis, which falls below the commonly accepted optimal threshold of 90% for *H. pylori* eradication therapies. While statistically non-inferior, this absolute efficacy level is approximately 3 percentage points lower than that of the VBQ regimen (90.4%). Therefore, the clinical application of the VD regimen should be carefully considered. It may serve as a valuable alternative primarily for patients with amoxicillin allergy or intolerance, or for those who have failed previous amoxicillin-containing therapies. For patients without such limitations who prioritize the highest possible cure rate, regimens with proven efficacy above 90% remain preferable.

Subgroup analyses showed that, in treatment-naïve patients, both the VA and VD regimens achieved eradication rates comparable to the VBQ regimen across the ITT, mITT, and PP analysis sets, supporting the efficacy of dual therapy as a first-line treatment. Among rescue therapy patients, the VD regimen demonstrated an eradication rate of 88.9% (ITT), suggesting its potential value as a salvage option. In contrast, the VA regimen showed a lower eradication rate in the rescue subgroup, indicating that vonoprazan-amoxicillin dual therapy may be less reliable in patients with prior treatment failure. Notably, the subgroup analysis of rescue therapy patients was limited by the small sample size. Only 33 patients had a history of prior eradication treatment. This limited sample size resulted in wide confidence intervals for the risk difference estimates and reduced statistical power to detect true differences or to establish non-inferiority, particularly for the VA regimen. Therefore, the findings from the rescue therapy subgroup should be considered exploratory rather than confirmatory. Future studies with larger sample sizes specifically targeting rescue therapy patients are needed to validate the efficacy of vonoprazan-based dual therapy with either amoxicillin or doxycycline in this population.

This study also systematically evaluated the safety of the three treatment regimens. The results showed no statistically significant difference in the overall incidence of adverse events between the vonoprazan-low-dose amoxicillin dual therapy and the bismuth-containing quadruple therapy. In contrast, the overall incidence of adverse events was significantly lower in the vonoprazan-doxycycline dual therapy group compared to the bismuth-containing quadruple group. The incidence of adverse events did not exceed 25% in any of the three regimens. These findings suggest that dual therapies, particularly the vonoprazan-doxycycline regimen, offer a favorable safety profile while maintaining eradication efficacy. Analysis of specific adverse event types revealed no statistically significant differences between the vonoprazan-amoxicillin dual group and the bismuth-containing quadruple therapy group in the incidence of abdominal pain, diarrhea, dizziness, acid reflux/heartburn, nausea/vomiting, abdominal bloating, rash, or bad breath. This may be related to the fact that amoxicillin itself can cause gastrointestinal reactions to some extent (Li et al., 2025; Axiotakis et al., 2022). In contrast, the vonoprazan-doxycycline dual therapy demonstrated significant advantages across several adverse events, with patients in this group experiencing a significantly lower incidence of acid reflux/heartburn, abdominal bloating, and abdominal pain compared to the quadruple group. This can likely be attributed to multiple factors. First, the dual therapy does not contain bismuth, fundamentally eliminating the risk of bismuth-related adverse events. Second, doxycycline, as a tetracycline-class antibiotic, has a weaker irritant effect on the gastrointestinal tract compared to amoxicillin (Singh et al., 2021). Additionally, the use of fewer antibiotics reduces the potential for gut microbiota disruption, further contributing to the favorable safety profile.

Regarding medication adherence, all three groups demonstrated excellent adherence, exceeding 96%, indicating a high degree of treatment completion. Although the quadruple regimen was more complex, involving a higher daily dosing frequency, there was no statistically significant difference in adherence between the two dual therapy groups and the bismuth-containing quadruple therapy group. This may be attributed to the comprehensive medication guidance and follow-up provided to patients in this study. Nevertheless, from a clinical practice perspective, dual therapy simplifies the treatment regimen and theoretically offers advantages for improving long-term patient adherence, warranting its further promotion.

Against the backdrop of increasing emphasis on antimicrobial stewardship principles, the paradigm for *H. pylori* eradication therapy should shift from the traditional “addition” approach to a “subtraction” strategy. For many years, in response to the challenge of rising resistance rates, clinical practice has tended to rely on increasing the number of antibiotics, prolonging treatment duration, or elevating drug doses to ensure eradication efficacy. However, this strategy not only increases the risk of adverse events but also exacerbates the problem of antibiotic resistance. This study provides robust evidence supporting the subtraction strategy. On one hand, vonoprazan combined with low-dose amoxicillin dual therapy achieved eradication rates non-inferior to those of quadruple therapy, challenging the conventional notion that amoxicillin requires high-frequency dosing. On the other hand, the high eradication rate of vonoprazan-doxycycline dual therapy suggests that antibiotic selection need no longer be limited to amoxicillin, offering a new option for patients with amoxicillin allergy or treatment failure. These findings indicate a promising future for dual therapy in *H. pylori* eradication. Antibiotic doses can transition from high to low, and antibiotic selection can expand from amoxicillin to other low-resistance drugs such as doxycycline. This not only aligns with the core principles of antimicrobial stewardship but also promotes a more precise, safer, and more sustainable approach to the treatment of *H. pylori* infection.

The exclusion criteria in this study were designed to minimize confounding factors while ensuring patient safety. Specifically, we excluded patients with confirmed active peptic ulcer disease to avoid potential confounding of adverse events, as such patients may present with disease-related symptoms (e.g., abdominal pain, bleeding) that could be misinterpreted as drug-related adverse events. Patients who had received antibiotics, bismuth agents, PPIs, or H_2_ receptor antagonists within the specified washout periods were excluded to eliminate the potential impact of prior medication use on *H. pylori* diagnosis and resistance profiles. Patients receiving concomitant medications such as corticosteroids, nonsteroidal anti-inflammatory drugs, anticoagulants, barbiturates, phenytoin, or carbamazepine were excluded due to the possibility of drug-drug interactions with the study medications. In addition, we excluded patients with severe comorbidities, those who were pregnant or lactating, and those with alcohol abuse to protect vulnerable populations and avoid safety concerns. Although these exclusion criteria are methodologically sound and enhance safety, they may limit the generalizability of our findings to real-world clinical practice. In routine clinical settings, patients with *H. pylori* infection often have comorbidities, take concomitant medications, or belong to special populations excluded from this study. Therefore, our findings apply primarily to generally healthy adults aged 18–70 years without active peptic ulcer disease, recent use of antibiotics or acid suppressants, or significant comorbidities. Caution should be exercised when extrapolating these results to populations excluded from the trial. Future studies specifically targeting these excluded populations are needed to further validate the safety and efficacy of dual therapy in broader clinical contexts.

The findings of this study should be interpreted in the context of regional differences in antibiotic resistance patterns and treatment guidelines. In China, resistance rates to clarithromycin and levofloxacin are high, whereas resistance to amoxicillin and doxycycline remains low (Zhong et al., 2021). Against this background, our results show that both vonoprazan-amoxicillin and vonoprazan-doxycycline dual therapies were non-inferior to bismuth-containing quadruple therapy in the Chinese population. However, extrapolation of these findings to Western populations requires careful consideration. 2024 ACG Clinical Guideline (Chey et al., 2024) recommend optimized bismuth quadruple therapy for 14 days as the preferred regimen when antibiotic susceptibility is unknown, with vonoprazan-amoxicillin dual therapy as an alternative empirical option for patients without penicillin allergy. The Maastricht VI/Florence consensus report (Malfertheiner et al., 2022) also emphasizes the importance of susceptibility testing in guiding treatment selection and notes the advantages of vonoprazan-amoxicillin dual therapy in infections with clarithromycin-resistant strains, while also highlighting that further optimization of eradication strategies is needed in non-Asian populations. In summary, although our study provides robust evidence for the efficacy of vonoprazan-low-dose amoxicillin and vonoprazan-doxycycline dual therapies in Chinese populations, differences in baseline characteristics, resistance profiles, and adherence patterns between Eastern and Western populations should be taken into account when extrapolating these findings to European and American populations. Such extrapolation should be based on local resistance data, guideline recommendations, and the generally lower eradication rates observed in Western trials.

This study has several limitations. First, although we explored multiple factors potentially influencing *H. pylori* eradication success using univariate and multivariable logistic regression analyses, no significant associations were identified. This may be attributed to several reasons. The relatively small sample size of the eradication failure group may have resulted in insufficient statistical power to detect true effects. Additionally, while some variables showed no statistically significant differences between groups, the absolute differences observed suggest that these trends might become significant with a larger sample size. Furthermore, data on *H. pylori* resistance testing were not included in this study. Antibiotic resistance is a critical factor affecting eradication efficacy, and its absence may have limited the comprehensiveness of our assessment. Future large-scale, multicenter prospective studies incorporating resistance testing are warranted to more accurately identify independent risk factors for eradication failure. Second, a visual assisted recall method was used to collect data on participants’ antibiotic use over the previous two years. While this approach may reduce recall bias compared to unaided verbal questioning, it cannot fully ensure accurate recollection of the exact timing, dosage, or duration of antibiotic use. Because the study did not have access to Electronic Medical Records or a Drug Utilization Review system for cross verification, the antibiotic use data may still be subject to recall bias, which could have influenced the analysis of related variables. Third, antibiotic susceptibility testing was not performed to characterize the distribution of *H. pylori* resistance in the study population. Therefore, we were unable to further elucidate the impact of uneven resistance distribution to amoxicillin and doxycycline on eradication outcomes. Fourth, the study was geographically limited to Jiangsu Province; therefore, the generalizability of the results to other regions in China or globally requires further validation. Fifth, in this study, adherence was assessed using paper record forms that participants completed daily during the 14-day treatment period and reported via online platforms or telephone follow-up within three days after treatment completion. While this method is more rigorous than retrospective recall alone, it still relies on patient self-report and may be subject to certain biases. First, participants might not have completed the record forms in real time every day as instructed, potentially leading to incomplete or inaccurate records. Second, the paper record forms could not independently verify whether the medications were actually ingested as recorded. Future studies should consider employing more objective adherence monitoring methods, such as pill counts, or electronic medication monitors to obtain more accurate and verifiable adherence data.

## 5 Conclusion

This study demonstrated that vonoprazan-low-dose amoxicillin dual therapy achieved non inferior eradication rates of approximately 90% compared to bismuth containing quadruple therapy, with favorable safety and simplicity, supporting its use as a reliable first line option for treatment naïve patients. The vonoprazan-doxycycline dual therapy also met statistical non inferiority criteria but achieved a lower eradication rate of approximately 87%, which falls below the optimal 90% benchmark. Therefore, the doxycycline based regimen should be interpreted with caution and may be considered primarily for patients with amoxicillin allergy or intolerance, balancing its modest efficacy against its favorable safety profile. Both regimens align with the core principle of antimicrobial stewardship, representing a shift from addition to subtraction by reducing antibiotic exposure and regimen complexity. However, further large scale multicenter studies are warranted to confirm the efficacy of the doxycycline regimen in diverse populations before it can be recommended for widespread clinical application.

## Data Availability

The raw data supporting the conclusions of this article will be made available by the authors, without undue reservation.
